# Non-Local Temporal Difference Network for Temporal Action Detection

**DOI:** 10.3390/s22218396

**Published:** 2022-11-01

**Authors:** Yilong He, Xiao Han, Yong Zhong, Lishun Wang

**Affiliations:** 1 Chengdu Institute of Computer Application, Chinese Academy of Sciences, Chengdu 610081, China; 2 School of Computer Science and Technology, University of Chinese Academy of Sciences, Beijing 100049, China

**Keywords:** temporal action detection, deep learning, convolutional neural networks, computer vision, video understanding

## Abstract

As an important part of video understanding, temporal action detection (TAD) has wide application scenarios. It aims to simultaneously predict the boundary position and class label of every action instance in an untrimmed video. Most of the existing temporal action detection methods adopt a stacked convolutional block strategy to model long temporal structures. However, most of the information between adjacent frames is redundant, and distant information is weakened after multiple convolution operations. In addition, the durations of action instances vary widely, making it difficult for single-scale modeling to fit complex video structures. To address this issue, we propose a non-local temporal difference network (NTD), including a chunk convolution (CC) module, a multiple temporal coordination (MTC) module, and a temporal difference (TD) module. The TD module adaptively enhances the motion information and boundary features with temporal attention weights. The CC module evenly divides the input sequence into N chunks, using multiple independent convolution blocks to simultaneously extract features from neighboring chunks. Therefore, it realizes the information delivered from distant frames while avoiding trapping into the local convolution. The MTC module designs a cascade residual architecture, which realizes the multiscale temporal feature aggregation without introducing additional parameters. The NTD achieves a state-of-the-art performance on two large-scale datasets, 36.2% mAP@avg and 71.6% mAP@0.5 on ActivityNet-v1.3 and THUMOS-14, respectively.

## 1. Introduction

With the wide application of image content understanding technology and the rapid growth of video data, video content understanding has attracted the attention of both industry and academia. It has application requirements in many scenarios, such as security surveillance, precision medicine, and video audits. One of the pressing needs is understanding human action within videos. Previous work tackled it as a pure action recognition task. In recent years, action recognition technology has made great achievements. However, action recognition requires trimmed videos with only action instances. In actual scenarios, most video data are unlabeled. Temporal action detection (TAD) can predict temporal boundaries (start/end) as well as action categories in an untrimmed video. Therefore, as an upstream task of video content analysis, TAD has become one of the bottlenecks that needs to be broken through.

In addition to the content information, the video also has contextual relevance, which requires modeling long-range temporal structures. The usual practice is to stack 1D convolutions. However, the original video has the following characteristics: (1) the duration of different actions varies widely; (2) information redundancy between neighboring frames; and (3) the information is weakened in the long-distance delivery. How to design an efficient network to model long-range temporal relationships while taking into account the above problems is the key to further improve the performance of a TAD task. In recent years, many works have tried to solve these problems, but most of them only consider one or two aspects.

To model long-range temporal dependencies, the commonly used methods are the stacked 1D temporal convolutions [1,2,3] and transformer [4,5,6]. However, limited by the kernel size, the former method can only capture the local scope context information, neither can learn the relationship between frames with distant temporal intervals, and it cannot establish the relationship between instances. Due to the redundant information of adjacent frames in a video, this method is prone to fall into local traps. With the success of transformers in object detection [7,8] and NLP [9,10], part work has migrated the self-attention mechanism to the temporal action detection task. The attention mechanism can learn the relationship between each frame and other frames one-to-one, avoiding the distance limitation of a 1D convolution. Because the length of the action instances is much smaller than the length of the video, such methods not only perform a lot of invalid computations but may also introduce irrelevant information. Therefore, neither the global nor the local scope can effectively model complex temporal dependencies. To solve this problem, we propose a chunk convolution (CC). Specifically, each chunk consists of three independent, traditional 1D temporal convolutions with fixed intervals, which not only enlarges the temporal receptive field but also alleviates the redundancy problem. In addition, a multi-branch strategy is adopted, where each branch handles a specific redundancy rate to be compatible with redundancy rate changes.

Similar to object detection, temporal action detection also belongs to the category of visual detection, which is to locate and classify potential objects. Object detection aims to generate bounding boxes in an image (2D), while temporal action detection aims to predict the boundary locations of action instances in a temporal sequence (1D). Therefore, most of the current methods for processing a temporal multiscale are migrated from an image multiscale. Considering that it is difficult to find a specific receiving field that balances all scales, TAL-Net [11], A2net [12], and DCAN [13] borrow the idea of an anchor in object detection, which consists of K-convolution blocks with parallel structures. Each block has a different kernel size, corresponding to a different temporal receiver field. The responses of all blocks are fused to provide finer-grained features. Due to the unsatisfactory effect of a large-size convolution kernel, such methods are not scalable enough. Inspired by res2net [14] and Xception [15], we propose a cascade residual architecture to process the temporal multiscale issue. Specifically, the module consists of several parallel branches, and each branch contains two 1D convolutions with kernel sizes of 1 and 3, respectively. Except for the first branch, the output features of the former branch are added with the input features as the input of this branch. Each time features pass through a branch, its temporal receptive field will expand once. Finally, the features from all the branches are concatenated along the temporal dimension to aggregate the features with different temporal receptive fields.

Among all the features, motion information and boundary features undoubtedly play an important role in precise locating. Some work [16,17] uses an optical flow to represent the motion information. However, as the network deepens, the motion information will weaken over a long-range delivery. In order to address the issues, we propose a temporal difference (TD) module. Concretely, the temporal-level action confidences are firstly calculated across the full sequence, where the scores represent the attention weights. These weights are then used to produce motion-sensitive weights. Finally, we utilize multiplication between the original feature and motion-sensitive weights to enhance the discriminability of the features. In this way, the network has the ability to adaptively discover and enhance the features of motion-sensitive locations. We proposed an NTD network that achieves a new state-of-the-art performance on two large-scale datasets, ActivityNet-v1.3 [18] and THUMOS-14 [19].

## 2. Related Work

Temporal action detection aims to classify and localize action instances in an untrimmed video as precisely as possible. The existing approaches can be divided into three main types: anchor-based, anchor-free, and the bottom–up method.

Anchor-based methods rely on manually pre-defined K anchors with different scales. The early anchor mechanism is a window anchor. The S-CNN [20] uses sliding windows to generate multiple candidate regions and then uses a binary classification network to identify a possible action instance. The TURN [21] and CTAP [22] first generate multiscale candidate regions at each temporal position and then use temporal regression to refine boundary positions. The window mechanism can cover all action instances, thus avoiding missed detection. However, the disadvantages are also obvious, generating a large number of redundant regions and the boundaries are imprecise. Inspired by a faster-rcnn [23] in object detection, the R-C3D [24] predicts the relative offsets and corresponding classification scores of K-different scales at each temporal position. Considering that the duration of the action instance varies more dramatically than the target in an image, the TAL-Net [11] proposes to align the temporal receiving field of the anchor with the corresponding temporal span. Manually defining the scales limits the ability to handle complex variations. The GTAN [25] introduces nonlinear temporal modeling, cascades multiple feature maps with different temporal resolutions, and learns a Gaussian kernel for each temporal position to predict the relative offset. The PBRNet [26] cascades three detection modules; the first module generates coarse results and subsequent modules further the boundary position.

Inspired by the successful application of the anchor-free detector in object detection, many methods adopt the anchor-free method, which directly predicts the boundary position without manually specifying the proposal scale. The AFSD [27] proposes a purely anchor-free framework that directly predicts the distance of the boundary (start and end) from each temporal position. However, the predicted proposal relies heavily on local information and does not make full use of context relations. In order to model long-range context, some current works, such as the RTD-Net [5] and TadTR [28], regard video as a temporal sequence and introduce a self-attention transformer structure. Because using the attention mechanism in the whole sequence is inefficient and will introduce irrelevant noise interference, ActionFormer [4] proposed a local attention mechanism that limits the attention range within a fixed window. Considering the anchor base and anchor free have the advantages of stability and flexibility, respectively, the A2net [12] integrates these two methods into one framework to achieve complementary advantages.

Bottom–up methods mainly focus on evaluating “probabilities”. The SSN [29] directly predicts the binary action probabilities for each frame in the video. Then, continuous frames with high action probabilities are grouped by the watershed algorithm to generate candidate proposals. The BSN [30], BMN [31], and BSN++ [32] predict the probabilities of being a start/end/action for each frame and then adopt a boundary-matching strategy to match pairs of start and end, generating candidate proposals with a flexible duration. These approaches fail to take full advantage of contextual information by focusing only on the confidence of isolated frames; this makes it sensitive to noise and prone to generating false positives and incomplete action instances. The BU-MR [33] exploits potential constraints between frame-level probabilities to provide more complementary information. The P-GCN [34] and G-TAD [35] take the proposals generated by the BSN as input and then use a graph convolution to explore the semantic relationships between proposals, providing more clues to facilitate boundary refinement.

## 3. Approach

### 3.1. Chunk Convolution

Given a sequence *X* ∈ $R^{C\times T}$, we evenly divide *X* into *N* = *T*/$\left(\omega +k\right)$ chunks, where *k*, *C*, and *T* denote kernel size, feature dimension, and temporal length, respectively, and ω is a manually set parameter used to adjust the chunk size. The *j*-th position of the *i*-th chunk can be represented as $\left(i,j\right)$, where *i* ∈ $\left[1,N\right]$, *j* $\in \left[1,\omega +k\right]$. When extracting features at position $\left(i,j\right)$, the difference from traditional 1D convolution is that in addition to applying standard 1D convolution here, 1D convolution block is also applied to adjacent chunks $\left(i-1,j\right)$ and $\left(i+1,j\right)$, respectively. Three convolution blocks form a chunk convolution, and the outputs of all convolution blocks are fused by summation as the output of the chunk convolution. To facilitate implementation, we transform the temporal dimension from 1D to 2D, *X* ∈ $R^{C\times T}$ → $X^{\prime }\in$ $R^{C\times N\times \left(\omega +k\right)}$, $\left(\omega +k\right)$ represents the temporal length of each chunk, and *N* represents the number of chunks. In order to keep the feature dimension constant, the operation of padding 0 around $X^{\prime }$ is adopted, $X^{\prime }\in$ $R^{C\times N\times \left(\omega +k\right)}$ → $X^{\prime \prime }$ ∈ $R^{C\times \left(N+2\right)\times \left(\omega +k+2\right)}$. Then, we apply 2D convolution to $X^{\prime \prime }$ as we did for extracting image features.

(1) $$H=W*X^{\prime \prime },\;H\in R^{C\times N\times \left(\omega +k\right)}$$

where ∗ represents the convolution operation, $W\in R^{C\times C\times K\times K}$ is the convolution kernel. Subsequently, the temporal dimension of *H* is restored from 2D to 1D, *H* $\in R^{C\times N\times \left(\omega +k\right)}\to Y_{CC}\in R^{C\times T}$, whose dimensions are consistent with the input features. Taking into account videos with various redundancy rates, we parallelize multiple branches with different chunk sizes to extract features simultaneously. In our experiments, the chunk size between d ∈ {4, 7, 9}, and the kernel size of all 1D convolutions is 3. It is easy to conclude that the dilate rates of the three branches are 1, 4, and 6, and the corresponding temporal receptive fields are 11, 17, and 21, respectively. The output of all branches is aggregated by max operation along the temporal dimension.

### 3.2. Multiple Temporal Coordination

**Figure 1:**
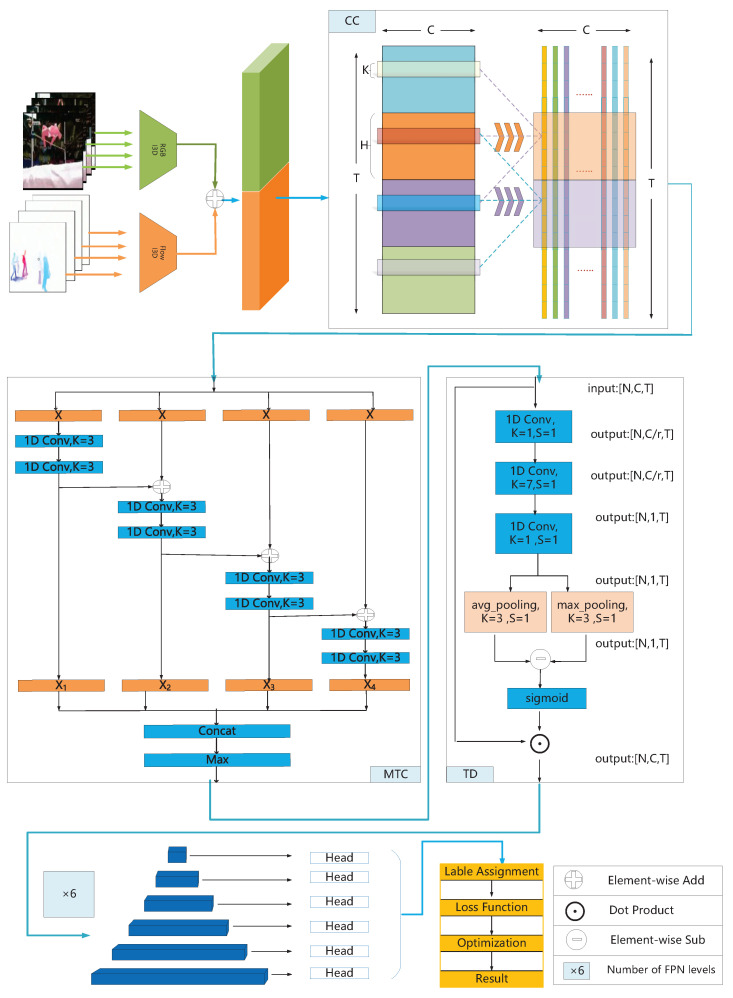
Overview of our proposed NTD. NTD first transforms the raw video into a sequence of clip features. Then, the clip features sequentially go through three modules. CC module, delivering information over long distances. MTC module, aggregating multiscale temporal features. TD module, adaptively enhancing motion information and boundary features. Finally, the encoded features pass through the temporal feature pyramid module, and the prediction head is responsible for generating detection results.

A simple and effective strategy to extend the temporal receiving field is to stack multiple 1D convolutional layers. However, the duration of the action instances in the videos vary significantly. We adopt a split–transform–merge approach to deal with multiscale problems. As shown in Figure 1, given an input feature $Z\in R^{C\times T}$, we feed it into four branches with identical structure. Each branch is composed of a 1D convolution with kernel size 1, followed by a 1D convolution with kernel size 3. The relationship between adjacent branches is transformed from parallel to cascade through residual connections. Thus, the output can be expressed as:

(2) $$F_{i}=W_{3}*\left(W_{1}*Z\right),\;i=1;$$

(3) $$F_{i}=W_{3}*\left(W_{1}*\left(Z+F_{i-1}\right)\right),\;i=2,3,4$$

where ∗ represents the convolution operation, $W_{1}$ and $W_{3}$ denote the 1D convolution with kernel sizes of 1 and 3, respectively. Here, *Z* is the input features and $F_{i-1}$ is the output features from the previous branch. The operation *Z* + $F_{i-1},i\in \left[2,4\right]$ is implemented by element-wise addition, where *Z* and $F_{i-1}$ have equal dimensions. Each convolution operation is followed by a nonlinear activation function Relu, which is omitted for simplifying formula. The function $W_{1}$ is used to learn the residual mapping, and $W_{3}$ is used to expand the temporal receptive field.Obviously, after the feature passes through a branch, the temporal receptive field will be enlarged one time. Moreover, except for the first branch, each branch aggregates the feature information from former branch. Therefore, this module not only expands the temporal receptive field but also aggregates features of different receptive field. The output of this module is a multiscale temporal feature set $\left\{F_{1},F_{2},F_{3},F_{4}\right\}$. Compared with the input feature *Z*, its temporal receptive field is enlarged by one, two, three, or four times, respectively. Finally, we adopted MAX operation along the temporal dimension on the set *S* to generate multiscale temporal features.

(4) $$Y_{\mathrm{MTC}}=MAX\left(\left[F_{1},F_{2},F_{3},F_{4}\right]\right),\;F_{i}\in R^{C\times T},Y_{\mathrm{MTC}}\in R^{C\times T}$$

### 3.3. Temporal Difference

There is no doubt that among all features, boundary features and motion information play a particularly important role in the accurate localization of action instance. However, the boundary points of action instances are relatively sparse, and motion information delivered from distant frames are weakened. Therefore, in order to solve the problem of information weakening caused by stacking convolution layers, it is necessary to find motion-sensitive temporal locations, and then enhance its features. We will implement it in two steps, squeeze and excitation. As depicted in Figure 1, given an input sequence $V\in R^{C\times T}$, in order to generate the temporal attention weights, we first consider using squeeze-channel operations. Specifically, stacking three 1D convolution layers, transform the feature dimension from $V\in R^{C\times T}$ to $G\in R^{1\times T}$. The first convolutional layer is used to reduce channel dimensions from *C* to *C*/*r*, *r* is set 4 in our work. Aims to capture long-range information, we followed a 1D convolution layer to extend receiving field, which set the kernel size as 7 and step size is 1 in our paper. The last 1D convolutional layer squeezes the channel dimensions into one. In addition, each convolutional layer is followed by an activation function Relu. In this way, we obtain temporal attention weights $G\in R^{1\times T}$ for each temporal position.

(5) $$G^{\prime }=\sigma \left(conv1*V\right),\;G^{\prime }\in R^{C/r\times T}$$

(6) $$G^{\prime \prime }=\sigma \left(conv7*G^{\prime }\right),\;G^{\prime \prime }\in R^{C/r\times T}$$

(7) $$G=\sigma \left(conv1*G^{\prime \prime }\right),\;G\in R^{1\times T}$$

where ∗ denotes convolution operation, σ refers to the Relu function, conv1 and conv7 indicate the 1D convolution whose kernel size is 1 and 7, respectively. We follow the squeeze operation with an excitation operation which aims to salient the boundary features. In practice, the attention weights between adjacent temporal location vary significantly, and this location can be approximately confirmed as a motion-sensitive position. It is achieved by feeding the attention weights into two independent branches, average pooling and max pooling, respectively. Among them, the difference calculation of the two branches can be formulated as:

(8) $$S=\delta \left(MAX\left(G\right)-AVG\left(G\right)\right),\;S\in R^{1\times T}$$

Here, δ represents the activation function Sigmoid, MAX and AVG denote max pooling and average pooling along the temporal dimension, respectively. Finally, the purpose of this module is to enhance boundary features and motion information; a straightforward way is to rescale features $V\in R^{C\times T}$ with the attention weights *S* $\in R^{1\times T}$.

(9) $$Y=V@S,\;Y\in R^{C\times T}$$

where @ refers to temporal-wise multiplication between the scalar *S* and feature *V*.

## 4. Training and Inference

### 4.1. Training

Before the encoded features are fed into the prediction layer, it goes through a six-level temporal feature pyramid module to be compatible with multiscale action instances. The hierarchical architecture is responsible for generating feature set M = $\left\{m_{1},m_{2},\cdots ,m_{6}\right\}$ with varying temporal resolutions. Precisely, we adopt the 1D convolution with stride s = 2 (except for the first level) to decrease the temporal length of each level. Our prediction layer consists of two independent lightweight convolutional networks for classification and regression, and both branches are implemented by three consecutive 1D convolutional with kernel size 3. Our network outputs the predicted result $y_{t}=\left(p_{t}^{i},d_{t}^{s},d_{t}^{e}\right)$ for every moment *t* across all pyramid levels. $p_{t}^{i}\in {\left\{0,1\right\}}_{1}^{c}$ is the probability of action categories (*c* pre-defined categories). $d_{t}^{e}$ ⩾ 0 and $d_{t}^{s}$ ⩾ 0 are the distance from the current moment *t* to boundary. $d_{t}^{s}$ and $d_{t}^{e}$ are valid if time *t* falls within the range of any action instances; otherwise, they are not counted as loss. In practice, the proportion of the background in the video is much higher than that of the foreground. To alleviate the imbalance, we adopt focal loss [36] as our classification loss function. According to the predicted classification score $p_{t}=\left(s_{0},s_{1},\cdots ,s_{c}\right)$, the total classification loss can be calculated using the following formula:

(10) $$L_{cls}=\frac{1}{T}\sum_{t=0}^{T}\sum_{i=0}^{c}-\alpha_{t}^{i}{\left(1-p_{t}^{i}\right)}^{\gamma }\log\left(p_{t}^{i}\right)$$

(11) $$\begin{array}{cc} p_{t}^{i} & =\left\{\begin{array}{cc} s_{i} & \;\mathrm{if}\;y_{t}^{i}=1,\;\;i=0,1,2,\cdots ,c\\ 1-s_{i} & \;\mathrm{otherwise},\;i=0,1,2,\cdots ,c \end{array}\right. \end{array}$$

(12) $$\begin{array}{cc} \alpha_{t}^{i} & =\left\{\begin{array}{cc} \alpha & \;\mathrm{if}\;y_{t}^{i}=1,\;\;i=0,1,2,\cdots ,c\\ 1-\alpha & \;\mathrm{otherwise},\;i=0,1,2,\cdots ,c \end{array}\right. \end{array}$$

In the above, class label $y_{t}\in R^{c}$ (*c* pre-defined categories), $y_{t}^{i}\in \left\{0,1\right\}$. γ and α are manually specified hyper-parameters, which are set to 2 and 0.25, respectively, in our paper. We use temporal Intersection over Union (tIoU) as the loss function for regression of the distance between the predicted instance ${\hat{\phi }}_{i}=\left({\hat{\psi }}_{i},{\hat{\xi }}_{i}\right)$ and the corresponding ground truth $\phi_{i}=\left(\psi_{i},\xi_{i}\right)$, and only the foreground moment that falls into an action instance is selected. The formula can be expressed as:

(13) $$L_{reg}=\frac{1}{T_{p}}\sum_{i}I\left(y_{i}\geq 1\right)\left(1-\frac{\left|{\hat{\phi }}_{i}\cap \phi_{i}\right|}{\left|{\hat{\phi }}_{i}\cup \phi_{i}\right|}\right)$$

where $T_{P}$ represents the number of foreground moments, the indicator function $I(y_{i}⩾1)$ is used to indicate whether the temporal location t∈[1,N] falls within the range of any ground truth.The total loss function employed during training is defined as follows:

(14) $$L=L_{cls}+L_{reg}$$

### 4.2. Inference

At inference stage, we directly input the feature sequences into the network. Our prediction layer outputs the predicted result $y_{t}=\left(p_{t}^{i},d_{t}^{s},d_{t}^{e}\right)$ for every moment t across all pyramid levels. For each moment t in the j-th pyramid level, the predicted action instance is denoted as

(15) $$a_{j}^{t}=\arg\max\left(p_{t}^{i}\right),\;s_{j}^{t}=t-d_{t}^{s},\;e_{j}^{t}=t+d_{t}^{e}$$

where $s_{j}^{t}$ and $e_{j}^{t}$ are the left and right boundaries of an action instance, and $a_{j}^{t}$ is the category score. Next, in order to remove the highly overlapping action instances, we aggregate the candidate action instances from all positions together, perform Soft-NMS [37], and obtain the final result.

## 5. Experiments

### 5.1. Datasets

We conduct the experiment on two popular benchmark datasets, ActivityNet-v1.3 [18] and THUMOS-14 [19], for the TAD task. THUMOS-14 collected videos of human daily activities, including 20 categories. The training and testing set contain 200 and 212 untrimmed videos, respectively. The average temporal length of the videos in the dataset is 4.4 min, each video contains more than 15 action instances, each instance has an average duration of 5 s, and more than 70% of the moments belong to the background. These action instances are densely distributed and disordered within the video, making it extremely challenging to perform TAD on this dataset. ActivityNet-v1.3 contains around 10 K, 5 K, and 5 K videos in the training, testing, and validation sets. As a larger dataset with 200 action categories, the average temporal length is 2 min, each video contains an average of 1.7 action instances, and each instance has an average duration of 48 s.

### 5.2. Evaluation Metrics

To compare with previous TAD methods, we adopt mean average precision (mAP) to evaluate our NTD network on both datasets. On THUMOS-14, the temporal Intersection-over-Union (tIoU) thresholds are selected from {0.3, 0.4, 0.5, 0.6, 0.7}. On Activitynet-v1.3, the tIoU thresholds are chosen from {0.5, 0.75, 0.95}. According to the official evaluation metrics, THUMOS-14 pays more attention to the performance on mAP@0.5, and Activitynet-v1.3 focuses on the results on mAP@avg [0.5:0.05:0.95].

### 5.3. Feature Extraction and Implementation Details

**Figure 2:**
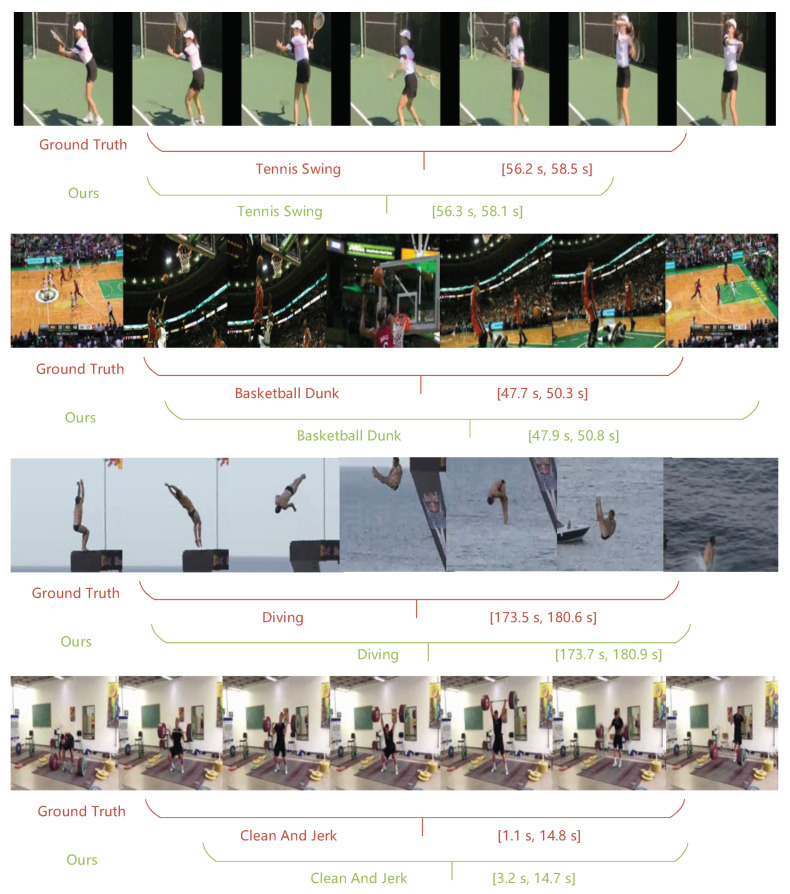
Qualitative Results. Visualize ground truth and corresponding predicted instances on THUMOS-14.

On THUMOS14, following [4,12], we adopt two-stream inflated 3D ConvNet (I3D [38]) module which pre-trained on Kinetics-400 [39] to extract spatial-temporal features from raw video. We sample 16 consecutive RGB and optical flow with the overlap rate of 75% as clips. Then, feed clip into I3D network and extract features of dimension 1024 × 2 at the first fully connected layer. Finally, the two-stream features are concatenated along the temporal dimension (1024D × 2 ⟹ 2048D). We use Adam [40] to optimize the network, setting the batch size, initial learning rate, total epoch number as 2, $1\times 10^{-4}$, 35, respectively. We visualize four instances predicted by our model on this dataset and compare them with the corresponding ground truth in Figure 2.

On Activitynet v1.3, following [4,12], we use R(2+1)D [41] pre-trained on TSP [42] to extract features. We sample 16 consecutive RGB with non-overlapping as clips. We use linear interpolation to rescale the feature sequence to a fixed length of 128. We use Adam to optimize the network, setting the batch size, initial learning rate, total epoch number as 16, $1\times 10^{-3}$, 15, respectively.

Our model is implemented based on PyTorch 1.1, Python 3.8, and CUDA 11.6. We conduct experiments with one NVIDIA GeForce RTX 3090 GPU, Intel i5-10400 CPU and 128 G memory.

## 6. Results

### 6.1. Comparison with State-of-the-Art Methods

We compare the NTD with several state-of-the-art temporal action detection methods on the THUMOS-14 dataset. As shown in Table 1, the performances at different tIoU thresholds (mAP@tIoU) vary from 0.3 to 0.7 as well as an average mAP [0.3:0.1:0.7] (mAP@avg). In comparison, our proposed NTD outperforms the other methods at all thresholds. In particular, under the official evaluation index mAP@0.5, our method achieves 71.6%, exceeding the concurrent state-of-the-art work of ActionFormer [4] by a large margin 6.0% (71.6% vs. 65.6%). We also achieve a state-of-the-art performance with an average mAP of 66.8% ([0.3:0.1:0.7]).

On the ActivityNet-v1.3 database, our model also achieves a competitive result, significantly outperforming the recent representative works, the AES [43], ActionFormer [4], BCNet [44], and RCL [1]; the performers are shown in Table 2. Our model achieves a 54.4% mAP@0.5, outperforming all of the previous methods. With an average mAP ([0.5:0.05:0.95]), our method reaches 36.3% which is 0.7% higher than the recent state of the art 35.6% by ActionFormer. This improvement is significant because the results are averaged over many tIoU thresholds, including those that are tight, such as tIoU = 0.95.

**Table 1:** Comparison with state of the art (THUMOS-14). We report the precision at different tIoU thresholds (mAP@tIoU) as well as average mAP in [0.3:0.1:0.7] (mAP@avg). The best results are in bold.

Method	Year	Backbone	0.3	0.4	0.5	0.6	0.7	AVG
S-CNN [20]	CVPR-2016	DTF	36.3	28.7	19.0	10.3	5.3	19.9
TURN [21]	ICCV-2017	Flow	44.1	34.9	25.6	-	-	-
R-C3D [24]	ICCV-2017	C3D	44.8	35.6	28.9	-	-	-
BSN [30]	ECCV-2018	TSN	53.5	45.0	36.9	28.4	20.0	36.8
TAL-Net [11]	CVPR-2018	I3D	53.2	48.5	42.8	33.8	20.8	39.8
GTAN [25]	CVPR-2019	P3D	57.8	47.2	38.8	-	-	-
P-GCN [34]	ICCV-2019	TSN	60.1	54.3	45.5	33.5	19.8	42.6
BMN [31]	ICCV-2019	TSN	56.0	47.4	38.8	29.7	20.5	36.8
A2Net [12]	TIP-2020	I3D	58.6	54.1	45.5	32.5	17.2	41.6
G-TAD [35]	CVPR-2020	TSN	54.5	47.6	40.2	30.8	23.4	39.3
BU-MR [33]	ECCV-2020	TSN	53.9	50.7	45.4	38.0	28.5	43.3
VSGN [45]	ICCV-2021	TSN	66.7	60.4	52.4	41.0	30.4	50.2
CSA [46]	ICCV-2021	TSN	64.4	58.0	49.2	38.2	27.8	47.5
AFSD [27]	CVPR-2021	I3D	67.3	62.4	55.5	43.7	31.1	52.0
MUSES [47]	ICCV-2021	I3D	68.3	63.8	54.3	41.8	26.2	50.9
RefactorNet [16]	CVPR-2022	I3D	70.7	65.4	58.6	47.0	32.1	54.8
ActionFormer [4]	2022	I3D	75.5	72.5	65.6	56.6	42.7	62.6
RCL [1]	CVPR-2022	TSN	70.1	62.3	52.9	42.7	30.7	51.7
AES [43]	CVPR-2022	SF R50	69.4	64.3	56.0	46.4	34.9	54.2
BCNet [44]	AAAI-2022	I3D	71.5	67.0	60.0	48.9	33.0	56.1
**NTD (Ours)**		I3D	**82.7**	**78.7**	**71.6**	**58.3**	**42.8**	**66.8**

The excellent performance demonstrates the effectiveness and generalizability of our proposed method for the TAL. This indicates that modeling long-range temporal context dependence while taking into account multiscale and motion information enhancement can improve the ability of the network to model complex video structures.

**Table 2:** Comparison with state of the art (ActivityNet-1.3). We report the precision at tIoU = 0.5, 0.75, and 0.95 (mAP@tIoU) as well as average mAP in [0.5:0.05:0.95] (mAP@avg). The best results are in bold.

Method	Year	0.5	0.75	0.95	AVG
TAL-Net [11]	CVPR-2018	38.2	18.3	1.3	20.2
BSN [30]	ECCV-2018	46.5	30.0	8.0	30.0
GTAN [25]	CVPR-2019	52.6	34.1	8.9	34.3
BMN [31]	ICCV-2019	50.1	34.8	8.3	33.9
BC-GNN [48]	ECCV-2020	50.6	34.8	9.4	34.3
G-TAD [35]	CVPR-2020	50.4	34.6	9.0	34.1
TCANet [49]	CVPR-2021	52.3	36.7	6.9	35.5
BSN++ [32]	AAAI-2021	51.3	35.7	8.3	34.9
MUSES [47]	CVPR-2021	50.0	35.0	6.6	34.0
ActionFormer [4]	2022	53.5	36.2	8.2	35.6
BCNet [44]	AAAI-2022	53.2	36.2	**10.6**	35.5
AES [43]	CVPR-2022	50.1	35.8	10.5	35.1
RCL [1]	CVPR-2022	51.7	35.3	8.0	34.4
**NTD (Ours)**		**54.4**	**37.4**	8.2	**36.2**

### 6.2. Ablation Study of MTC Module

In response to the problem that the duration of different actions varies widely, we design the multiple temporal coordination (MTC) module. In our experiments, different numbers of branches were tried, and the results are listed in Table 3. By comparing the first to sixth rows of the table, we can observe that with the increase in branches, the performance continues to improve. The best results, 71.6% mAP@0.5 and 66.8% mAP@avg, were obtained when the number of branches is four. However, the fifth and sixth rows show that using more branches does not achieve a better performance. In the last two rows, we also show the effectiveness of different feature fusion strategies between branches. By comparison, the MAX operation works best. This benefits from the feature selectivity of the MAX function, which improves the saliency of the feature maps within the regions. With each additional branch, the equivalent temporal receptive field will be enlarged one time. A multi-branch cross-scale association is beneficial to capture the multiscale feature information, but the scale span is significantly different, which will affect the stability of the module to capture local features.

### 6.3. Ablation Study of TD Module

We study the effects of a temporal receptive field (convolution kernel size) for the TD module on THUMOS-14, as shown in Table 4. Comparing the first to third rows, it shows that the TD benefits more from larger kernel sizes (*K* = 7 vs. *K* = 3). However, as the convolution kernel continues to expand, the mAP@0.5 drops by more than 1%. We also compared the effect of the max pooling size on the results when computing attention weights, and the results show that a smaller size (*S* = 3) performs better. Larger convolution kernels mean that the TD can capture the contextual information in a longer temporal range, thus mitigating random noise interference. However, an excessively large convolution kernel will smooth the difference between neighboring features.

### 6.4. Ablation Study of CC Module

We compare the performances of the aggregating chunk convolution features with different dilation rates, and the results are shown in Table 5. As can be observed, aggregating chunk convolutional features with a larger dilation rate generally yields a higher mAP. However, as the dilation rate continues to increase, it resulted in a performance degradation. In addition, we also compared the replacement of the ordinary 1D convolution with a dilated 1D convolution and did not obtain a better performance. This shows that taking into account different redundancy rates helps to improve the generalization of the model, but an excessive dilation rate hinders the capture of adjacent information, resulting in insufficient features information.

### 6.5. Ablation Study of Combination Strategies

In order to verify the effect of the three independent modules working together, the CC, MTC, and TD, we tried a variety of combination strategies, and the results are listed in Table 6. Obviously, the best performance is achieved when the combined strategy is CC → MTC → TD. Compared with the three-paths parallel mode, its performance exceeds at least 1.2% (mAP@0.5). In addition, it also has at least a 0.9% (mAP@0.5) advantage compared with other series combination strategies. This suggests that the optimal strategy is to take three steps. The CC module not only establishes long-range context dependencies but also effectively alleviates the local information redundancy. The MTC module and the following lightweight TD module are responsible for providing multiscale information and enhanced motion information. The three modules work together to better model the complex video structure.

### 6.6. Qualitative Results

Figure 2 visualizes the localization results and predicted categories of four action instances on THUMOS14 and compares the predicted results (green) with the corresponding GT (yellow). These instances include short (first and second), medium (third), and long (fourth) durations. It can be observed that the middle (third) and long (fourth) instances were correctly localized. However, the boundary positions of the short-duration instances (first and second) were imprecise. The reason lies in two aspects: the motion of a short instance changes rapidly, and the lack of contextual relevance makes it difficult to provide sufficient clues for the prediction layer. In addition, relative to the long instance, its IoU is extremely sensitive to the offset, so it is easy to be judged as a negative sample.

## 7. Conclusions

In this paper, we introduce a novel network for the temporal activity detection (TAD) task in untrimmed videos. Our proposed model consists of three modules that process input features in a serial manner. Specially, the input features are first passed through the CC module to reduce the redundant content while capturing long-range contextual information. Then, the output features of the previous step are processed by the MTC to aggregate the multiscale local features. Finally, the aggregated features are input to the TD module to enhance the weakened motion information and boundary features. Benefiting from the complementarity of three independent modules, our model outperforms the state-of-the-art methods by a big margin on two large-scale benchmarks, ActivityNet-v1.3 and THUMOS-14. Extensive experiments demonstrate the generalization ability and effectiveness of our approach.

Discussions: Temporal action detection is still an extremely challenging task, where the complexity of the video structure is an important factor. So far, it is still unclear how to effectively model complex temporal structures. The video has contextual relevance, which requires modeling long-range temporal structures. The usual practice is to stack 1D convolutions. However, the original video has the following characteristics: (1) the duration of different actions varies widely; (2) information redundancy between neighboring frames; and (3) the information is weakened in the long-distance delivery. According to the above characteristics, we designed the MTC, CC, and TD modules, respectively. The experimental results show that each module can help to improve the performance of the model. In addition, the video also has the characteristics of overlap, nonlinearity, spatio-temporal correlation, an inconsistent motion rate, and sparse boundary points. How to design an efficient network to model long-range temporal relationships while taking into account the video characteristics is the key to further improve the performance of the TAD task.

## Figures and Tables

**Table 3:** Ablation Study (impact of MTC module). Comparing the effects of different branch numbers and fusion strategies between branches on THUMOS-14, measured by mAP@tIoU at different thresholds and the average mAP (mAP@avg) [0.3:0.1:0.7].

Number	Strategy	0.3	0.4	0.5	0.6	0.7	AVG
6	MAX	81.3	77.3	69.9	59.4	43.2	66.2
5	MAX	81.2	77.3	70.0	58.2	44.4	66.2
4	MAX	82.7	78.7	71.6	58.3	42.8	66.8
3	MAX	81.7	77.8	71.2	60.0	44.9	67.1
2	MAX	81.6	78.0	70.5	57.7	43.2	66.2
1	MAX	81.0	77.0	69.4	59.0	43.8	66.1
4	AVG	81.9	77.9	69.8	57.2	42.7	65.9
4	Conv1D	81.8	77.3	70.1	58.9	44.4	66.5

**Table 4:** Ablation Study (impact of TD module). Comparing the effects of convolution kernel size (*K*) and max pooling size (*S*) on THUMOS-14, measured by mAP@tIoU at different thresholds and the average mAP (mAP@avg) [0.3:0.1:0.7].

*K*	*S*	0.3	0.4	0.5	0.6	0.7	AVG
3	3	82.1	77.5	71.2	58.0	43.3	66.4
5	3	82.1	78.4	71.3	59.6	43.4	66.9
7	3	82.7	78.7	71.6	58.3	42.8	66.8
9	3	81.1	77.3	70.5	57.7	44.4	66.2
11	3	81.6	77.2	70.6	58.3	43.8	66.3
7	5	81.9	77.9	70.2	58.1	43.4	66.3
7	7	81.2	77.3	70.5	58.4	43.7	66.2

**Table 5:** Ablation Study (impact of CC module). Comparing the effect of chunk convolutions with different dilation rates (D). DC represents dilated convolution, SC represents standard convolution, measured by mAP@tIoU at different thresholds and the average mAP (mAP@avg) [0.3:0.1:0.7]. √ indicate the selected dilation rate.

SC	DC	D = 1	D = 3	D = 6	D = 9	D = 13	0.3	0.4	0.5	0.6	0.7	AVG
√		√					81.7	77.5	70.9	58.5	43.3	66.4
√		√	√				82.1	78.0	70.3	57.6	43.4	66.3
√		√	√	√			82.7	78.7	71.6	58.3	42.8	66.8
√		√	√	√	√		81.7	77.2	69.7	57.9	42.6	65.8
√		√	√	√	√	√	81.4	77.8	70.8	58.7	43.2	66.4
	√	√	√	√			82.1	77.9	70.5	58.5	43.9	66.6

**Table 6:** Ablation Study (impact of combination strategies). Comparing the effects of different combined strategies, measured by mAP@tIoU at different thresholds and the average mAP (mAP@avg) [0.3:0.1:0.7].

Strategy	0.3	0.4	0.5	0.6	0.7	AVG
CC → MTC → TD	82.7	78.7	71.6	58.3	42.8	66.8
CC → TD → MTC	81.9	77.7	70.3	57.6	42.5	66.0
TD → CC → MTC	81.8	77.8	70.4	58.3	42.5	66.2
TD → MTC → CC	81.7	77.6	70.0	56.8	43.2	65.8
MTC → TD → CC	81.9	78.1	70.7	57.7	43.5	66.4
MTC → CC → TD	82.2	77.6	70.4	58.7	43.7	66.5
Stack (avg)	82.2	77.9	70.4	57.8	43.5	66.3
Stack (max)	82.0	77.7	70.1	58.2	43.7	66.3
Cancat	81.1	76.9	69.8	58.3	44.0	66.0
